# Forensic analysis of suicide deaths: Comparing forensic information with public information and investigating factors contributing to psychiatric consultations

**DOI:** 10.1002/pcn5.194

**Published:** 2024-04-24

**Authors:** Ryu Murakami, Atsushi Kamikubo, Daigo Morioka, Hisanaga Kuroki

**Affiliations:** ^1^ Faculty of Emergency Medical Science Meiji University of Integrative Medicine Nantan Kyoto Japan; ^2^ Graduate School of Risk and Crisis Management Chiba Institute of Science Chiba Japan; ^3^ Osaka Prefectural Medical Examiner's Office Osaka Japan

**Keywords:** forensic medicine, medical examiner, psychiatric consultation, suicide deaths

## Abstract

**Aim:**

This study aimed to examine the usefulness of forensic information on suicide deaths in Japan for epidemiological studies on suicide and determine the factors that lead people with suicidal ideation to seek psychiatric care prior to attempting suicide.

**Methods:**

We focused on forensic information of 514 suicide deaths that occurred in Osaka City in 2019. First, to examine whether the data used in this study can be generalized to these cases, we compared the information on suicide deaths officially published by Osaka City with that used in this study, utilizing Pearson's *χ* ^2^ test. Next, the forensic data were analyzed using multivariate logistic regression analysis to confirm the relationship between demographic factors and the likelihood of having a history of psychiatric consultation before suicide.

**Results:**

Both the official information and the data used in this study showed a higher number of males than females who died by suicide, with no significant differences in values between the data sets. Multivariate logistic regression analysis showed significant correlations. More females were associated with seeking a psychiatric consultation. However, those with regular jobs or students were more likely to avoid a psychiatric consultation.

**Conclusion:**

The findings of this study provide insights into the phenomenon of suicide deaths by using a forensic perspective. The results of this study suggest that psychiatric consultation may be effective in reducing deaths by suicide. Forensic data should be incorporated into the formulation of suicide‐prevention policies in Japan to conduct a more multifaceted analysis and improve suicide‐prevention measures.

## Graphical abstract

This study aimed to examine the factors that led individuals to seek psychiatric consultation before suicide. Analysis revealed that females were more likely to consult than males. However, those in regular employment or students showed a lower likelihood of having prior psychiatric consultations compared with unemployed individuals. The results of this study suggest that psychiatric consultation may be effective in reducing suicide deaths.

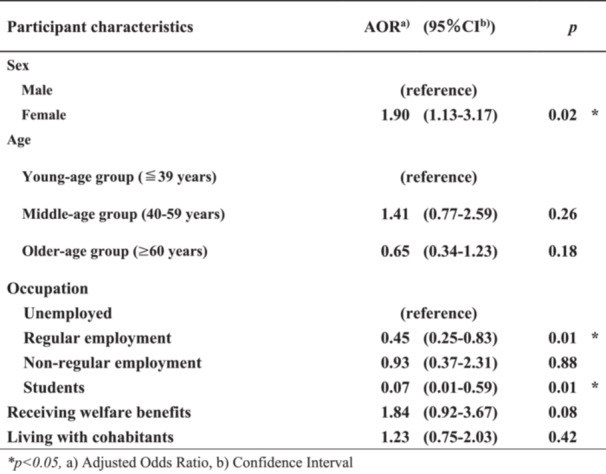

## INTRODUCTION

Japan has the highest suicide mortality rate among the Group of Seven (G7) countries. In particular, suicide is the leading cause of death in the age group deeply involved in production, including those within an age range of 10–34 years old.^1^ There are two official sources of information on suicide deaths in Japan, which are gathered from national surveys: the data collated from the police department's original suicide statistics record (hereafter “suicide statistics”) and the vital statistics on population dynamics from the Ministry of Health, Labour, and Welfare (hereafter “population dynamics”). Suicide statistics cover suicide deaths by both Japanese people and foreigners (including tourists) in Japan. When the police determine through their investigation that the cause of death is suicide, an officer creates a record for the suicide statistics, and the suicide deaths are then counted based on the location (prefecture) where the deceased was found. By contrast, the population dynamics target suicide deaths of Japanese nationals and are recorded based on the deceased's residential registration address. If, after being initially categorized as an “unknown cause of death,” a subsequent correction report clarifies the death as a suicide based on the death certificate or similar documents, it is retroactively counted. Disparities in various statistical data on suicide have been discussed both domestically and internationally.^2^, ^3^, ^4^ A study conducted by Takizawa that examined the mental disorders in individuals who died by suicide pointed out discrepancies between population dynamics and suicide statistics in terms of reported suicide deaths associated with mental disorders.^4^ The disparities in the base data of these statistics pose challenges for in‐depth studies on the background of deaths by suicide. In practice, “suicide” is categorized as a nonnatural death (i.e., death not caused by underlying diseases during treatment). The police conduct investigations, including those on the deceased's body. In cases in which individuals were transported to medical institutions because of a nonfatal attempt at suicide and later died, an investigation by the police or an examination by a physician is performed. A death is determined as a “suicide” only after any connections between the deceased's death and crimes or other external factors are negated. Multiple agencies, including the police, are involved before a death is classified as a “suicide,” which makes it challenging to approach and trace the deceased's medical history, including any prior conditions. Research on the backgrounds of those who have died by suicide, including psychological analysis of family members of the deceased, and studies that include deaths by suicide have been conducted both in Japan and abroad.^5^, ^6^, ^7^, ^8^, ^9^, ^10^ These studies have played vital roles in revealing the reality of suicide deaths. However, obtaining consent from participants is essential for psychological autopsies, and it is difficult to generalize insights from cases of nonfatal suicide attempts to those who have died by suicide. The World Health Organization has mentioned that suicide data collected from specific medical institutions is largely influenced by the hospital's record‐keeping process. This includes the potential for the repeated inclusion of individuals with more than one suicide attempt in 1 year or cases where individuals were treated in an emergency department but were discharged before formal admission and therefore not included in reports.^11^ Osaka City, the research area for this study, is designated under the medical examiner system. For nonnatural deaths in which the possibility of a crime‐related death is determined to be low by the police, medical examiners perform examinations and autopsies to elucidate the cause of death. Article 8 of the Body Autopsy Preservation Act classifies bodies as those with suspicions of death by infectious diseases, poisoning, or disasters and those for which the cause of death is unclear, including suicides. The information used in this study is detailed in documents (hereafter “target documents”) that the police send to the medical examiner when requesting an examination. These documents provide detailed accounts of suicides identified during police investigations and include information obtained from inquiries to related organizations, such as hospitals and local governments. This can include a psychiatric medical history, cohabitation, and other factors. Notably, in 2019, the research data covered Osaka City, which has the second‐largest population in Japan following Yokohama City^12^ and is a region under the medical examiner system. Thus, all suicide deaths are included, except for the judicial autopsy cases that occurred in Osaka City. This may minimize the potential for selection bias compared with using information collected at a specific medical institution. Conducting a detailed analysis of suicide information in target documents and linking it to elucidate the reality of suicide is highly beneficial from a public health perspective. Prior studies^7^, ^13^, ^14^, ^15^, ^16^, ^17^, ^18^ identified a connection between suicide and mental disorders. Thus, clarifying this relationship is critical for suicide prevention. To obtain epidemiological information on suicide deaths, it is important to obtain information on suicide deaths as well as on persons who have attempted suicide. However, in Japan, there is no nationwide organization to investigate the cause of death, and medical universities and administrative agencies in each prefecture are currently responsible for investigating the cause of death. To the best of our knowledge, no study in Japan has examined factors related to a history of psychiatric consultation from a forensic perspective based on information on suicide deaths. In this study, we extracted information on suicide deaths from target documents maintained by the Osaka Prefectural Medical Examiner's Office and examined the relationship between suicide and psychiatric disorders. In particular, we examined the usefulness of epidemiological studies using information on individuals who died by suicide obtained from a forensic perspective and the factors associated with psychiatric consultation prior to death by suicide. This manuscript is a statistical analysis of a paper presented at the 119th Annual Meeting of the Japanese Society of Psychiatry and Neurology.

This study aimed to identify factors contributing to the psychiatric consultations of individuals prior to their deaths by suicide using information on those who died by suicide, as recorded in documents delivered to the Osaka Prefectural Medical Examiner's Office by the police.

## METHODS

### Study subjects and duration

This study used anonymized information from 514 suicide cases that occurred between January 1, 2019, and December 31, 2019, in the city of Osaka and were listed in target documents. Target documents include one sheet of paper for each case, containing a summary of the nonnatural deaths. Each item used in this study is identified by an entry field (age) and a check box (gender, occupation, welfare benefits, living situation, and psychiatric consultations) in the target documents. We transcribed these items from the target documents into electronic files and used them as statistical information. The Osaka Prefectural Medical Examiner's Office provided approval for reviewing these documents.

### Comparison between Osaka Prefecture's published figures and the study documents

As a preliminary survey, the number of suicides reported by the Osaka Prefecture (hereafter, “published figures”) and those recorded in target documents were compared to verify the validity of the suicide investigations based on the information in target documents. Pearson's *χ* ^2^ test was used to determine the difference in the number of suicides between the published figures and target documents. A multiple comparison test was used to evaluate the differences between each item in the published figures and target documents.

### Extraction of factors related to psychiatric consultation history before death by suicide

Multivariate logistic regression analysis was conducted to identify factors associated with psychiatric consultations prior to death by suicide and to examine the relationship between psychiatric consultation history and the backgrounds of individuals who died by suicide.

### Data items

#### Comparison between Osaka Prefecture's published figures and the target documents

For the published figures, the study referred to information on suicide cases discovered in Osaka City, as reported in the “Breakdown of Suicides in Each Municipality in the First Year of Reiwa (confirmed values).”^19^ The comparison was based on (i) sex (a. male, b. female), (ii) age (a. young‐age group, b. middle‐age group, c. older‐age group; classification was based on a previous study^5^), (iii) occupation (a. unemployed, b. regular employment, c. non‐regular employment, d. students, e. unknown), (iv) welfare benefits (a. receiving, b. not receiving, c. unknown), and (v) living situation (a. with cohabitants, b. without cohabitants, c. unknown). As the published figures did not mention welfare benefits, this item was excluded from the verification process.

#### Extraction of factors related to psychiatric consultation history before death by suicide

The primary evaluation item was the presence or absence of psychiatric consultation history; the explanatory variables included (i) sex (a. male, b. female), (ii) age (a. young‐age group, b. middle‐age group, c. older‐age group), (iii) occupation (a. unemployed, b. regular employment, c. non‐regular employment, d. student), (iv) welfare benefits (receiving or not receiving), and (v) living situation (with or without cohabitants). All suicide data in this study pertained to the deceased individuals. This included cases where the deceased was in an advanced state of decomposition upon discovery or where, although the individual was identified, the circumstances immediately before death were unknown (under investigation) at the time of police information provision to the medical examiner. Cases with missing values for either the primary evaluation item or explanatory variables were excluded.

### Statistical analysis

The Bonferroni method was used to correct *p*‐values for multiple tests. The significance level was set at *α* = 0.05 (two‐sided), and a result was considered statistically significant if *p* < 0.05. The confidence intervals were two‐sided, with a confidence coefficient of 95%. All statistical analyses were performed using IBM SPSS Statistics (Ver.29.0.0.0; IBM).

## RESULTS

### Comparison of the published figures and target documents

Among the suicide cases that occurred in Osaka Prefecture in 2019, we conducted a Pearson's *χ* ^2^ test on the difference in the number of suicides between the published figures and the target documents for those found in Osaka City, which was the survey area (Table 1).

**Table 1:** Comparison of Osaka Prefecture published figures and target documents.

	Published figures		versus	Target documents		
*n* (%)	449	(100%)		514	(100%)	*p* a
Sex						0.74
Male	295	(65.7%)		343	(66.7%)	
Female	154	(34.3%)		171	(33.3%)	
Age						0.64
Young‐age group (≦39 years)	117	(26.1%)		148	(28.8%)	
Middle‐age group (40–59 years)	166	(37.0%)		184	(35.8%)	
Older‐age group (≥60 years)	166	(37.0%)		182	(35.4%)	
Occupation						<0.01*
Unemployed	295	(65.7%)		311	(60.5%)	
Regular employment	110	(24.5%)		127	(24.7%)	
Non‐regular employment	27	(6.0%)		39	(7.6%)	
Student	16	(3.6%)		16	(3.1%)	
Unknown^b^	0	(0.0%)		21	(4.1%)	
Welfare benefits						**NA**
Receiving	NA			80	(15.6%)	
Not receiving	NA			379	(73.7%)	
Unknown	NA			55	(10.7%)	
Living situation						<0.01*
With cohabitants	239	(53.2%)		242	(47.1%)	
Without cohabitants	210	(46.8%)		237	(46.1%)	
Unknown^b^	0	(0.0%)		35	(6.8%)	

* *p* < 0.05.

^a^ Pearson's *χ* ^2^ test.

^b^ Bonferroni's multiple comparison test was conducted to determine statistically significant categories between the published figures and the surveyed subjects.

Regarding sex in both data sets, although males outnumbered females, no statistically significant difference was observed between the published figures and the target documents in the number of suicides (*p* = 0.74). For age, no statistically significant difference was observed between the two documents in any age group (*p* = 0.64). For occupation, a statistically significant difference was observed (*p* < 0.01). Therefore, we conducted a multiple comparison test for the number of suicide deaths in each occupation category between the published figures and target documents, revealing a statistically significant difference in the unknown group. A statistically significant difference was observed in the living situation category (*p* < 0.01). A multiple comparison test for the number of suicides in each living arrangement category between the published figures from Osaka Prefecture and the target documents showed a statistically significant difference in the unknown group.

### Extraction of factors associated with the history of psychiatric consultation before death by suicide

**Figure 1:**
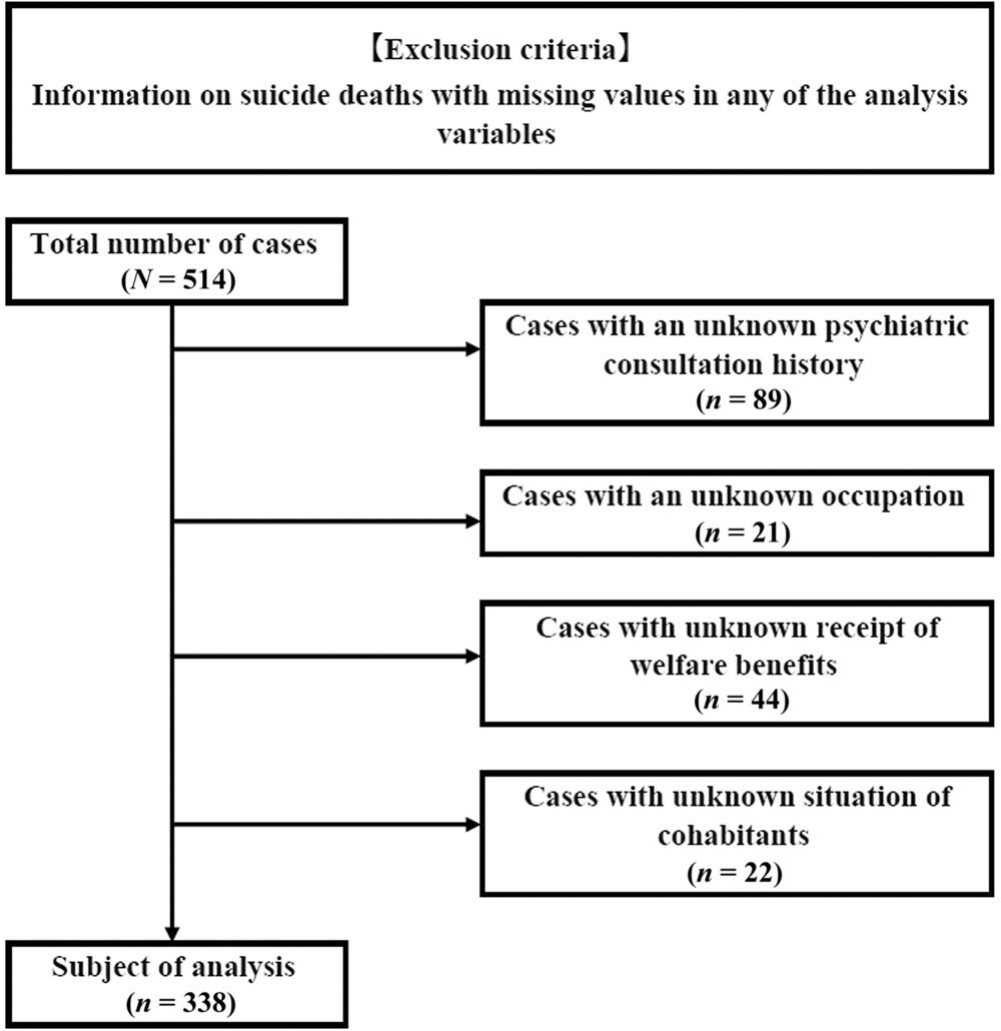
*Exclusion criteria*: In conducting a multivariate logistic regression analysis, cases with missing values in either the main evaluation items or explanatory variables of the subjects were excluded. From the total number of cases (*n* = 514), the following were excluded: (1) cases with unknown psychiatric consultation history (*n* = 89), (2) cases with unknown occupation (*n* = 21), (3) cases with unknown welfare benefits reception (*n* = 44), and (4) cases with unknown cohabitant living situations (*n* = 22). The remaining cases (*n* = 338) were used for the analysis.

In the multivariate logistic regression analysis, we excluded cases with missing values for any of the main evaluation items or explanatory variables (Figure 1). Table 2 lists the breakdown of the survey subjects after excluding the missing values. Regarding sex, males (63.0%) outnumbered females (37.0%), and among those with a history of psychiatric consultations, there were more females (44.9%) than males. Regarding age, the young‐age group was the smallest (29.3%) and the middle‐age group was the largest (37.6%), and in the group with a history of psychiatric consultations, the middle‐age group remained the largest (43.4%). Regarding occupation, the unemployed group was the largest (65.7%), followed by the regular employment group (23.4%), the non‐regular employment group (7.7%), and students (3.3%). Moreover, the number of students without a history of psychiatric consultation (7.0%) was higher than that of students with a history of psychiatric consultation (0.5%). Regarding welfare benefits, the majority did not receive welfare benefits (81.7%)—only approximately 18.3% of the sample received them. Regarding living situation, the number of those living without cohabitants (52.4%) was slightly higher than that of those living with cohabitants (47.6%). Next, we present the results of the multivariate logistic regression analysis, with the presence or absence of a psychiatric consultation history as the main evaluation item (Table 3). The independent variables adjusted for each explanatory variable and showing a significant association with the presence or absence of a psychiatric consultation history were as follows: sex—female compared with male (odds ratio [OR]: 1.87, 95% confidence interval [CI]: 1.12–3.12), occupation—regular employment compared with unemployed (OR: 0.47, 95% CI: 0.26–0.87), and occupation—student compared with unemployed (OR: 0.05, 95% CI: 0.01–0.44).

**Table 2:** Identification of factors related to psychiatric consultation by individuals who died by suicide.

	Total number		Psychiatric consultation history			
				Yes		No
*n* (%)	338	(100%)	196	(58.0%)	142	(42.0%)
Sex						
Male	213	(63.0%)	108	(55.1%)	105	(73.9%)
Female	125	(37.0%)	88	(44.9%)	37	(26.1%)
Age						
Age of average	50.77	(SD = 19.1)	51.3	(SD = 17.9)	50.1	(SD = 20.6)
Young‐age group (≤39 years)	99	(29.3%)	51	(26.0%)	48	(33.8%)
Middle‐age group (40–59 years)	127	(37.6%)	85	(43.4%)	42	(29.6%)
Older‐age group (≥60 years)	112	(33.1%)	60	(30.6%)	52	(36.6%)
Occupation						
Unemployed	222	(65.7%)	145	(74.0%)	77	(54.2%)
Regular employment	79	(23.4%)	34	(17.3%)	45	(31.7%)
Non‐regular employment	26	(7.7%)	16	(8.2%)	10	(7.0%)
Students	11	(3.3%)	1	(0.5%)	10	(7.0%)
Welfare benefits						
Receiving	62	(18.3%)	43	(21.9%)	19	(13.4%)
Not receiving	276	(81.7%)	153	(78.1%)	123	(86.6%)
Living situation						
With cohabitants	177	(52.4%)	106	(54.1%)	71	(50.0%)
Without cohabitants	161	(47.6%)	90	(45.9%)	71	(50.0%)

**Table 3:** Results for the multivariate logistic regression analysis, with the presence of a psychiatric consultation history as the main evaluation item.

Participant characteristics	AOR	(95％ CI)	*p*
Sex			
Male	(reference)		
Female	1.90	(1.13–3.17)	0.02*
Age			
Young‐age group (≦39 years)	(reference)		
Middle‐age group (40–59 years)	1.41	(0.77–2.59)	0.26
Older‐age group (≥60 years)	0.65	(0.34–1.23)	0.18
Occupation			
Unemployed	(reference)		
Regular employment	0.45	(0.25–0.83)	0.01*
Non‐regular employment	0.93	(0.37–2.31)	0.88
Students	0.07	(0.01–0.59)	0.01*
Receiving welfare benefits	1.84	(0.92–3.67)	0.08
Living with cohabitants	1.23	(0.75–2.03)	0.42

Abbreviations: AOR, adjusted odds ratio; CI, confidence interval.

* *p* < 0.05.

## DISCUSSION

In this study, by targeting the information handled by both the police officers responsible for the initial response to suicide cases and the medical examiner who investigates the causes of death from a forensic perspective, it was suggested that background information on suicide deaths within the realm of forensic medicine can be utilized as a new information source for epidemiological surveys on suicide deaths. We believe that it is essential to consider the relationship between the background factors of those who died by suicide and the act of suicide itself to understand the mechanism and risk factors of suicide.

### Comparison of the published figures and target documents

Pearson's *χ* ^2^ test was conducted on the number of suicide deaths mentioned in the published figures versus the target documents, revealing statistical significance in occupation and living situation. For both categories, statistical significance was observed for the “unknown” items upon conducting multiple comparison tests for each item (Table 1). Regarding the number of suicide deaths for these items, both indicate zero cases as “unknown” in the published figures. The published figures are recorded as final figures for the respective year, and the foundational data for these numbers are obtained from the “Basic Documents on Suicides in the Region,” created by the Ministry of Health, Labour, and Welfare, based on the original suicide death statistics from the National Police Agency. Suicide cases treated as nonnatural deaths have been identified over time through police investigations, and consolidated data have been gathered by the National Police Agency. The published figures represent the finalized figures, whereas the information described in our study's target documents is a preliminary report sent to the Osaka Prefectural Medical Examiner's Office by the police soon after the discovery of a death by suicide (or nonnatural death). Thus, there is a time difference between the two sources when they aggregate their data after the discovery of a death by suicide. This variation might have caused the difference in the results of the “unknown” item between the published figures and the target documents, which is believed to be the cause for the observed statistical significance. The target document delivered to the medical examiner focuses on nonnatural deaths within Osaka City, especially those discovered in ambiguous boundary areas, such as rivers, where the document reaches the medical examiner. These cases may have caused discrepancies in the number of suicide deaths between the published figures and the target documents. Nevertheless, because there are no statistically significant differences between the published figures and the target document in terms of sex and age group, as well as because the published figures do not include a history of psychiatric consultation—the item that is the focus of this study, and therefore cannot be investigated—the target document is considered worthy of study.

### Breakdown of suicide deaths in this study

Of the 514 documents studied, we categorized 338 cases, excluding those with missing values, based on their psychiatric consultation history (Table 2). The occurrence of missing values is believed to be influenced by the timing of data collection for the deceased. The details of suicide deaths have gradually become clear through investigations by the police and medical examiners. However, given that the target documents are sent to medical examiners as preliminary reports during investigations of nonnatural deaths, some information remains unclear. Nevertheless, it is of substantial significance to conduct epidemiological surveys on suicide deaths based on information verified alongside the bodies of the deceased by both the police and medical examiners. It is crucial to use this research as a stepping‐stone for further investigations on individuals who have died by suicide. The multivariate analysis of suicide deaths (Table 2) shows that males accounted for 63.0% of the individuals who died by suicide. According to suicide statistics from 2019, the sex composition of suicides nationwide included 69.8% males. This result aligns with those of previous studies,^20^, ^21^, ^22^ indicating more suicide deaths among males than females. When looking at the psychiatric consultation history based on sex, females had a higher rate of consultations. Prior research has indicated a gender paradox in suicidal behavior,^9^, ^23^, ^24^, ^25^, ^26^ with men dying by suicide more frequently while women are recorded as having a history of significantly greater nonfatal suicide attempts. In this connection, traditional masculine gender norms are often mentioned as a factor in the reluctance to seek emotional support.^27^, ^28^, ^29^, ^30^ Thompson et al.^31^ report that gender differences in healthcare‐seeking behavior and the extent of primary care visits were higher for women than for men, both physically and mentally. Hirai et al.^32^ examined factors that led to successful mental health care visits, indicating that the main reason for the visit was subjective norms, such as being recommended by others to see a mental health care provider. In addition, several studies have suggested that positive attitudes toward treatment by family members or partners and encouragement to seek mental health care from close relatives may have a positive impact on patients' chances of receiving mental health care.^33^, ^34^, ^35^, ^36^, ^37^, ^38^ For example, a family member or partner who discovers self‐injury or trauma recommends that the patient could visit an emergency room, and the physician refers the patient to a psychiatrist; thus, witnessing self‐injury may lead to medical support for the patient. The middle‐aged group was recorded as having a higher history of psychiatric consultation than other age groups. A systematic review reporting on factors related to suicide access to mental health services found that men and individuals belonging to younger or older age groups were associated with nonaccess to mental health services.^39^, ^40^ In addition, women and individuals over 50 years of age had higher rates of access to mental health services. However, this finding differs from the findings of Hirokawa et al.,^5^ which were used as a reference for age‐group classification in conducting this study (their study had the highest percentage of young adults). Since few studies have compared the factors involved in the rate of psychiatric consultation among individuals who died by suicide by age group, and the methods of age‐group classification vary among studies, it is difficult to simply compare the results among studies. In the future, it may be desirable to establish a uniform data‐collection method in the field of epidemiological studies on suicide deaths, on a national and possibly global scale, with respect to age‐group‐based comparisons.

### Multivariate logistic regression analysis

Understanding the characteristics of patients with a history of psychiatric consultations who have died by suicide can contribute to more proactive medical interventions for high‐risk patients in psychiatric settings. From the results of the multivariate logistic regression analysis among individuals who died by suicide, being female was a factor associated with psychiatric consultation before death. This could be related to the previously mentioned gender paradox in suicidal behavior. Among individuals who died by suicide, compared with being unemployed, being regularly employed or a student was a factor associated with not seeking psychiatric consultation. Regularly employed full‐time employees and students may experience more instances of resistance to consulting a psychiatrist due to a variety of interactions in their environment (e.g., concerns about deteriorating friendships with colleagues or friends caused by stigma against psychiatric consultation and mental illness). The stigma associated with mental illnesses,^32^, ^41^, ^42^, ^43^ such as depression or schizophrenia, could influence their decision to not seek psychiatric help. Access to mental health professionals is crucial for preventing suicides due to mental illnesses.^44^, ^45^, ^46^ Therefore, eliminating the stigma toward mental illnesses can be effective in suicide prevention. The results of this study provide foundational data for promoting consultations in psychiatry.

### Limitations

This study has several limitations. Although the data aggregated all suicide cases occurring in Osaka City, thereby ensuring a comprehensive local sample, the findings might lack generalizability beyond this specific region. The application of multivariate logistic regression, although rigorous, might not have captured all influencing factors or confounding variables. Furthermore, the cross‐sectional nature of our approach could have overlooked the evolving personal factors that influence psychiatric consultations.

## CONCLUSIONS

Based on the background information of suicide deaths, we investigated the factors contributing to psychiatric consultations from a forensic perspective. Among those who died by suicide, being female was associated with having a history of psychiatric consultation before death. Moreover, compared with being unemployed, being a regular employee or student was identified as a factor related to not having a history of psychiatric consultation. Given the sex and occupational differences in the results of this study, psychiatric consultations may have had a suppressive effect on deaths by suicide. These results, derived purely from the background information of those who have died by suicide, can deepen our understanding of suicide prevention.

We believe that the formulation of suicide‐prevention measures is an urgent issue in Japan. In fact, as part of a national effort to prevent suicide, the Comprehensive National Suicide Prevention Plan^47^ includes items for promoting suicide‐prevention measures at the national and municipal levels. Among the items to be promoted is a section on “Suicide investigation for children, young people, and women, and linkage with the system for determining the cause of death.” However, we believe that investigation of suicide deaths in the field of forensic medicine, including the cause‐of‐death investigation system, is currently at a developmental stage. In the results of this study, factors associated with a history of psychiatric consultation were identified as those linked with deaths by suicides. The results could be used, for example, to interview patients who are in contact with psychiatric consultation in terms of suicide intervention. We also believe that the results will serve as a foundation for selecting items to be collected in the future when obtaining medical statistics with a primary focus on suicide research. It is important that the fields of forensic medicine, psychiatry, sociology, and other academic disciplines collaborate with practitioners in the national and regional government to develop a national strategy against suicide. In addition, we believe that the establishment of a professional and integrated statistical survey format for suicide research will improve suicide‐prevention measures.

We aimed to use the information in documents for suicide investigations from a forensic perspective, combined with investigative methods from other fields, including psychological autopsies, to conduct a more multifaceted analysis. A multidisciplinary collaboration can be considered the key to deepening our understanding of suicide prevention.

## AUTHOR CONTRIBUTIONS

Ryu Murakami was in charge of conducting the research and writing the paper. Atsushi Kamikubo collected research data, provided forensic advice, and revised the paper. Daigo Morioka conducted statistical analysis of the research data and interpreted the results, and Hisanaga Kuroki oversaw and managed the entire research project.

## CONFLICT OF INTEREST STATEMENT

The authors declare no conflicts of interest associated with this manuscript.

## ETHICS APPROVAL STATEMENT

This study was approved by the Meiji University of Integrative Medicine Human Ethics Committee (Receipt number: 2022‐039).

## PATIENT CONSENT STATEMENT

This study was performed by opt‐out procedures. To ensure that the opt‐out procedure is carried out in accordance with the procedures under the national ethical guidelines, the opt‐out document, which is prepared to allow the patient's family to request exclusion from the study, is posted on the website of the institution where the first author is affiliated. The print version of the document is available at the designated location of the author's facility.

## CLINICAL TRIAL REGISTRATION

N/A

## Data Availability

The data used in this study are based on documentary information stored in the Osaka Prefectural Medical Examiner's Office. Therefore, this information is not publicly available. Furthermore, to conduct this study, we used materials that have been processed to ensure that individuals are not identifiable. Statistical processing for this study was performed at an information terminal shielded from the Internet and external connections. The data used are stored on encrypted storage media and are strictly managed within the research institute to which the first author belongs.
